# Turbans vs. Helmets: A Systematic Narrative Review of the Literature on Head Injuries and Impact Loci of Cranial Trauma in Several Recreational Outdoor Sports

**DOI:** 10.3390/sports9120172

**Published:** 2021-12-20

**Authors:** Dirk H. R. Spennemann

**Affiliations:** Institute for Land, Water and Society, Charles Sturt University, Albury, NSW 2640, Australia; dspennemann@csu.edu.au

**Keywords:** outdoor recreational sports, recreational accidents, cranial trauma, turbans, helmets

## Abstract

When in public, faith-based mandates require practising Sikh men to wear a turban which may not be covered by hats or caps. This makes it impossible for practising Sikhs to wear helmets and other protective headwear, mandatory in many countries and facilities for engagement in recreational pursuits (e.g., skiing) and on adventure outdoor recreation camps mandatorily run for school groups. The result is often social exclusion and ostracisation in the case of school children. Despite studies into the efficacy of protective helmets in some recreational outdoor activity settings, virtually nothing is known about the protective potential of turbans. This paper systematically reviews the extant literature on head injuries in several recreational outdoor activities and sports sectors (aerial, water, winter, wheeled and animal-based sports) and finds that the extant literature is of limited value when trying to understand the spatial distribution of trauma on the cranial surface. As the data do not permit to make inferences on the protective potential of turbans, future systematic, evidence-based epidemiological studies derived from hospital admissions and forensic examinations are required. Failure to do so perpetuates social exclusion and discrimination of religious grounds without an evidentiary basis for defensible public health measures.

## 1. Introduction

Participation in recreational and professional sport is meant to be an all-inclusive activity, subject only to personal motivation and ability. Clearly social constraints, and some cultures’ gender-stereotyping, create extraneous influences that limit participation. In other settings, well-meaning legislative constraints to wear protective gear may impact participation, as the stipulations of the legal mandate clash with cultural norms. One of these incidences is the conflict between the culturally and spiritually mandated requirement to wear a turban and the legally mandated requirement to wear a helmet when engaging in a range of sports. A strict interpretation and application of the legal mandate effectively pose a stark choice for male Sikhs: violet the spiritual mandate to wear a turban or abstain from the sport. This paper examines evidence whether turbans might provide sufficient protection and thus could be worn in lieu of helmets.

Evidence on the protective potential of turbans as an alternative to safety helmets is very limited. Given that most Sikhs live in India, particularly Punjab, it is not surprising that the majority of anecdotal reporting on the role of turbans to limit or prevent cranial trauma comes from there. A review of the literature discussing cranial trauma of individuals not wearing helmets in the construction sector found that studies failed to detail the exact location of the head trauma, making it impossible to extract data that might constructively inform whether areas unprotected by turbans are disproportionately affected [1]. Likewise, a review of over 200 journal articles discussing cranial trauma in two-wheeler accidents on the Indian subcontinent found that while most studies on head injuries pass comment on the presence or absence of helmets worn by the riders and pillion passengers, the extent of the protection provided by a turban is either simply assumed or noted as a possible mitigating factor of unknown benefit, but has not been formally assessed [2]. An exception is a single evidence-based study by Sood, which found that the turban may have some degree of protection in minor motorcycle accidents [3].

### Social Relevance of the Narrative Review

All practising Sikhs are forbidden to cut their hair and trim or shave their beard. Among men, the hair is twice daily combed towards the front, where it is twisted and tied into a top knot *(joora*) that is positioned at the front of the head and commonly held in place with a comb. The hair must be covered to avoid impurities, using a piece of cloth (*patka*) in private and a turban in outdoor and formal settings [4]. Turbans and unshorn hair are an outward symbol of the Sikh faith and are important marks of a Sikh male’s identity. Decorative turbans, usually wound from a one-meter wide and five-meter long piece of lightweight fabric, have brought Sikhs in perpetual conflict with dress standards imposed by corporations or governmental instrumentalities in many countries worldwide [5]. While many of these conflicts could be resolved on the grounds that they violate the freedom of religious expression, the mandatory wearing of protective headwear (‘hard hat’, ‘helmet’) is a more intractable matter. According to Sikh religious practices, Sikhs may not cover their hair by any form of headwear other than a turban, and a turban may not be covered by any additional head coverings placed on top of it [5]. This practice effectively prevents practising Sikh men not only from wearing caps and hats but also from wearing protective helmets (as they are considered a form of a cap). This dissonance sets up a potential for conflict between the cultural expectations of Sikh men and the occupational health and safety regulations promulgated by individual companies and organizations or national legislation. This dissonance also plays out in the arena of adventure outdoor recreation activities [5].

Since the late 19^th^ century, Sikhs have emigrated to the diaspora, in particular to Australia, New Zealand, and Canada [6,7,8,9], and within the past generation, that immigration has accelerated. Australian census data, for example, show that the population of Sikhs increased from 26,500 in 2006 to 72,300 in 2011 and 125,000 in the 2016 census [10,11], with a high proportion of school-age children and young adults. With the increasing prevalence of adventure outdoor recreation activities at mandatory school camps and school-run resilience and personal development programs [12,13,14], Sikh children following their faith are effectively excluded from participation, and in consequence, often ostracized in their year cohort.

A review has shown that the national occupational health and safety regulations are not uniform but dependent on national or subnational jurisdiction [5]. In Canada, for example, Sikh males wearing a turban are exempt from the requirement to wear motorcycle helmets in the Canadian provinces of Alberta, British Columbia, Manitoba, and Ontario. Yet, such exemptions do not extend to construction sites unless protective headwear can be worn over an under-turban or *patka*. In several states of Australia, Sikhs wearing turbans are exempt from the mandatory wearing of bicycle helmets, but not from wearing helmets while riding motorcycles. Where a turban can legally be worn *instead of* protective headgear, the risk is uniformly and without recourse transferred to the turban wearer who has to accept full and sole responsibility for his decision and the impact it may have on his health or life [5]. 

In several jurisdictions, it is mandatory to wear helmets while engaged in outdoor sports, which leads to the exclusion of and nonparticipation by Sikhs in these sports. In the absence of specific studies that assess the efficacy of turbans in reducing the impact on the head during falls and collisions, we must rely on proxy data. This paper systematically canvasses and reviews the extant literature on head injuries in several recreational outdoor activities and sports sectors (aerial, water, winter, wheeled and animal-based sports). Where stated, data on impact loci are extracted and correlated against those regions protected or buffered by the turban.

## 2. Methodology

This paper systematically reviews the extant literature on head injuries in several recreational outdoor activity sectors. The methodology espoused a combination of systematic database searches with snowballing [15,16,17]. The relevant databases (SCOPUS, Primo, FACTIVA, MEDLINE, GoogleScholar, etc.) were systematically searched with the following keyword combinations: ‘head injury’|‘cranial fracture’ + ‘outdoor/adventure recreation’|(name of sport). Excluded were studies of head injuries sustained by motorized outdoor sports such as ATVs, dirt bikes and motocross, bungee jumping, backyard sports such as trampolines, or by participation in various team sports such as ice hockey, soccer, or cricket.

In total, 367 sources were identified from the following recreational pursuits: climbing (21), mountain biking (22), scooters (19), skateboarding (21), skating (22), equestrian (47), hang-gliding and paragliding (30), skydiving (16), BASE jumping (9), surfing (32), wind and kitesurfing (16), white-water kayaking and rafting (11), downhill skiing (33), cross-country skiing (11), snowboarding (40), sledding (14) and curling (3). 

**Figure 1:**
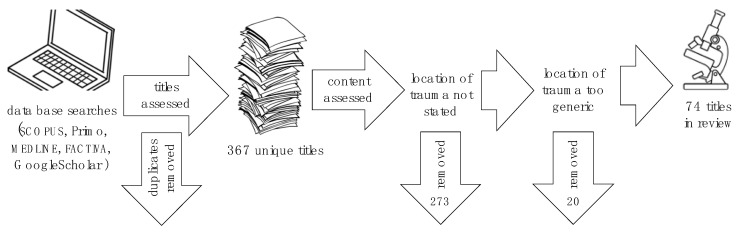
Workflow of review.

From these, 273 sources were discarded as the exact location of the trauma was not specified, and another 20 were removed as the location of the trauma was expressed in broad terms unsuitable for the analysis of impact loci required for this review (Figure 1).

### 2.1. Injury Locus Classification

**Figure 2:**
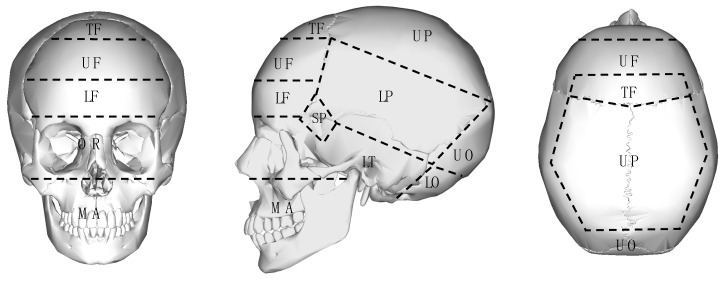
Schematic representation of impact zones on the skull. Abbreviations: Basal (B) (not shown), Lower Frontal (LF), Lower Occipital (LO), Lower Parietal (LP), Lower Temporal (LT), Maxillo–Mandibular (MA), Orbital (OR), Sphenoid (SP), Top Frontal (TF), Upper Frontal (UF), Upper Occipital (UO), Upper Parietal (UP.) (Base image of the skull: Wikimedia).

**Figure 3:**
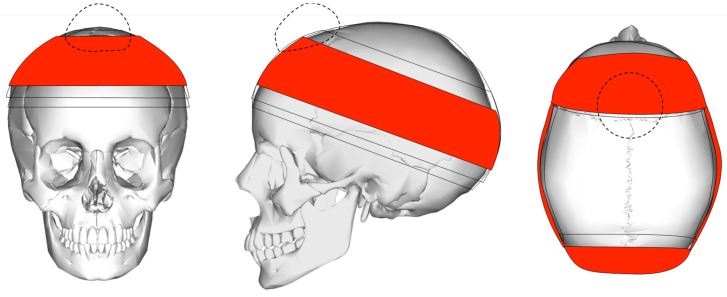
Schematic protection provided by a turban. The darker, the more layers. The dashed outline shows the approximate location of the joora, which provides additional buffering [2].

In order to approximate the extent of possible protection provided by a turban in case of sports-incurred head injury, the cranium was divided into several impact zones (Figure 2) based on a schematic coverage and the protection provided by a turban (Figure 3). As already noted, the majority of the studies included in this review do not identify the exact location of the head trauma, or else they only express it in broad terms that are unsuitable for the analysis of impact loci. Those studies that comment on the locus of injury classify it by the major bones making up the human cranium, limiting their suitability in this review.

Tominaga et al. calculated the cranium average surface area, noting that the two parietal bones make up 52% of the cranium, followed by temporal bones (including the sphenoids) with 23%, the frontal bone with 18%, and the occipital bone with 7% [18]. Combining these estimates with the extent of areas covered by a turban (Figure 3) suggests that a turban protects about 62% of the cranial surface. 

### 2.2. Impact Attenuation

The impact on the skull will be attenuated by the number of layers of turban cloth wrapped over that specific part of the cranium, as well as the style of the turban and the nature and thickness of the turban fabric. There are five main types of turbans ranging from the *Domalla* to the *Keski*. The *Domalla*, for example, is a round style, double-length turban using ten or more metres of fabric, while the *Keski* is a short turban of two or more metres, shaped similar to the *Domalla* but shorter and thus less thick. The most commonly worn is the *Pag(ri)*, which is a double-width (and thus double thickness) turban of five to six metres in length [5].

## 3. Results: Traumatic Head Injuries in Various Outdoor Sports

There is a growing interest in the nature and impact of head injuries across a wide range of outdoor sports, driven partly by the need to raise awareness of their potentially severe and debilitating effects and partly to improve or assess currently available or potential preventative and therapeutic measures for dealing with such injuries. However, the majority of the overview studies on head injuries associated with outdoor sports lack specificity as to the exact location of the head trauma. Given that the focus of this review lies in the spatial distribution of cranial trauma and the protective potential of turbans, these studies can provide only minimal support to our analysis. 

### 3.1. Rock and Ice Climbing

While falls make up 77% of all severe climbing-related injuries [19,20], head injuries are a major cause of severe trauma [21] and fatalities [22,23,24]. The causality of head trauma, where specified, is primarily being hit or struck by an object [19,25] or by falling ice while ice climbing [26]. 

While helmets provide some protection against falling objects, this is not universal. At present, there are no studies that examine the relative protection provided by helmets.

### 3.2. Wheeled Sports

#### 3.2.1. Mountain and Trail Biking

There are a plethora of studies that consider head trauma among cyclists. Since wearing bicycle helmets is compulsory in many jurisdictions, only a few studies assess head injuries among riders without helmets [27,28,29]. Given the plethora of literature on the topic, road cycling has been excluded from this review and shall become the focus of a separate study.

While head and neck injuries are more common among road (16%) vs. mountain bike (off-road) riders (6%) [30], mountain bikers experience considerable head acceleration during the pursuit of their activity [31] While helmets will lower the impact of an injury [32], they do not entirely prevent head them [33,34]. Comparisons of the incidence of head and neck injuries between amateur and the professional and elite level of the sport have shown inconsistent results [35,36].

On a generic level, loss of control during mountain biking tends to result in a forward motion with riders falling over the handlebars, in particular those with lighter bodyweight [37]. This type of fall differentially impacts the face and the frontal bone [38], with a high incidence of maxillar and mandibular fractures [37,39,40].

#### 3.2.2. Scooters

There are two types of scooters: unpowered, small-wheel push scooters and the more recently introduced electric scooters. Motorized, petrol-powered scooters (e.g., Vespas), which are sit-down devices and deemed a class of motorcycle, are excluded from analysis, as are mobility scooters.

Given the inherent stability of the small-wheel scooter design, small obstructions and uneven ground can result in a loss of control which causes the majority of accidents [41]. Most injuries resulted from falls forward or to the side, with head injuries being a major cause of severe trauma [42,43]. Reported trauma incidence incurred on push scooters ranged from 16.5% among adults to over 33% among children [42,43]. Riders of electric scooters had a higher incidence of head injuries, ranging from 36% to 42.7% [44,45]. Single case reports document a sideways fall with parietal trauma [46], a backwards fall resulting in the opening of the lambdoid suture [47].

#### 3.2.3. Skateboarding

Setting aside a small sample with no reported head injuries [48], the frequency of head injuries incurred during skateboard accidents ranges from 4% to almost 75% of all incidents [18,49]. Two-wheeled, self-balancing ‘hoverboards’ fall into the same range [50]. Several authors noted that head injuries occur more frequently when longboards are used [51,52], which can be attributed to an emphasis on overall speed. 

Tominaga et al. observed that 59.6% of all head injuries were incurred among people years 15 and over, with skateboarding injuries occurring in the occipital area, followed by temporal (17.7%) and frontal (14.9%) (*n* = 141) [18]. Only 5% of those admitted with head injuries wore a helmet. Given that the occipital bone makes up only 7% of the surface area of the cranium, the authors found the difference to be statistically highly significant.

#### 3.2.4. Skating

Given the etiology of the injuries, head injuries incurred while ice skating will also be addressed in this section. Excluded from this discussion, where possible, are in-line and roller-skating injuries incurred in collisions with moving or stationary traffic (‘dooring’). Observed head and face injuries in ice, in-line and roller skating tend to be low. Observed rates range from 0% to 11.8% for in-line skating [49,53,54,55] to almost 40% in ice-skating [55,56].

Ice skating children and adolescents showed a higher incidence of head and face injuries than adults, with 34.4% [56], while adults aged 50 and over had a higher incidence of head and face injuries than adults aged 18–50, 29.4% and 13.7%, respectively. The higher incidence among older participants is caused by loss of balance due to increased degradation of the inner ear. Children under seven years of age had the highest incidence of 56.3% [56]. The high representation of head injuries in children under seven years of age was also observed in other studies [55]. This may be explained in terms of lack of control due to limited experience as well as biomechanics, as children have a higher centre of gravity than adults [55,56].

There are two main types of falls, both caused by lack of balance and a consequent lack of control, seen in forward and backward falls. Video analysis of falls showed that the majority are forward and downward falls, irrespective of whether the sport involved ice skating or in-line skating (56.9% vs. 56.7%), followed by backwards falls (41.7% vs. 39.3%). Sideways falls were comparatively rare (1.4% vs. 4.0%) [57]. Given the prevailing direction of falls, head and facial injuries tend to exceed cranial trauma in all types of skating [55,58], which also suggests that among cranial trauma, the frontal region is disproportionately affected. There are differences in the kind of sport, however, with ice skating having by far the highest percentage of head and face injuries (13.3%, 26.6%) followed by in-line skating (5.0%, 6.8%), and roller skating (4.4%, 5.2%) [55].

### 3.3. Aerial Sports

#### 3.3.1. Hang-Gliding and Paragliding

Most injuries associated with paragliding (using a steerable parasail) and hang-gliding (using a rigid-framed wing) are associated with landing and thus manifest themselves in injuries to the lower extremities. Until the introduction of mandatory wearing of helmets, head trauma was not uncommon, however, especially among powered hang-gliders, but no locational data were reported.

#### 3.3.2. Sky-Diving 

Overall, head injuries are rare in skydiving, as the etiology of most trauma relates to injuries incurred to the lower extremities and spine while landing. The incidence of head trauma is higher among military parachutists [59,60], possibly due to additional gear and different jump conditions. Given the biomechanics of most rough landings, however, trauma to the occipital region can be assumed in most instances.

#### 3.3.3. BASE Jumping 

BASE jumping involves a short free fall followed by a parachute descent from tall structures or cliffs (Building, Antenna, Span, Earth). Given the low jump-off height (compared to skydiving) of only 150–200 m, a BASE jumper can collide with the ground or fixed object due to lack of control (due to environmental and equipment factors), which contributed to a 5× to 16× risk for death or injury when compared with skydiving [61]. 

### 3.4. Winter Sports

The etiology of accidents in snow sports varies, with falls predominating in skiing and snowboarding, while collisions with objects and other participants predominate in sledding and tubing sports [62,63,64]. Head injuries are the primary cause of death on ski slopes regardless of the type of snow sport [65,66] and tend to be more frequent among snowboarders than skiers [67,68,69]. The incidence of head injuries is related to the risk-taking behaviour of the participants. A study in the USA noted that head injuries are more frequent in terrain parks, which are more challenging but also induce participants to attempt jumps and stunts, than on standard slopes [70] At least one study noted that ‘research is needed to describe the nature of head injuries in skiers and snowboarders’ [71].

#### 3.4.1. Downhill Skiing

Observed head injuries in downhill skiing ranged from 15.7% to 38% [62,72], with experienced skiers being more represented, possibly due to a higher propensity to take risks [68]. 

The etiology of injuries sees forward falls as the primary cause of head injury, followed by sideward falls [64,73]. Only four studies detailed the location of the cranial injury and the type of fall, even though it is limited to the major bones (frontal, parietal, temporal, occipital, and facial) [64,73,74] Three of these have a larger sample that allows a more nuanced analysis [64,74,75]. Nakaguchi, when studying head injuries on Japanese ski slopes, found that trauma to the occipital area dominated (53.2%), followed by injuries to the frontal (37.3%) region [75]. Fukuda et al., also studying head injuries on Japanese ski slopes, found similar patterns, where that trauma to the occipital area dominated (40.8%), followed by injuries to the frontal (37.0%) and temporal region (18.5%) [74]. 

One study examined the location of the cranial injury and the type of fall [64]. Not surprisingly, forward falls, caused by loss of control when striking unevenness in the skiing surface or when crossing skis, predominantly impacted the frontal (36.9%) and facial bones (27.4%), followed by the occipital area (22.6%). Sideways falls, when catching ski edges, show a similar pattern, with injuries to the occipital area being 30.8% more frequent than facial trauma (26.9%). Falls caused by losing balance and falling backwards, not surprisingly, have the most significant impact on the occipital region (57.1%), with other regions (except for temporal) more or less equally represented. Injuries caused by collisions with other users or objects exhibit a more or less similar distribution of facial (30.5%), frontal (28.4%), and occipital trauma (27.7%). Temporal regions were the least impacted in all types of falls and collisions [64].

#### 3.4.2. Cross-Country Skiing

Observed head injuries in cross-country skiing are low, in the range of 5% to 10% [76]. The exact location of the head trauma is not specified in most studies. The etiology of injuries in cross-country skiing shows falls which tend to be forward falls resulting in facial injury [77].

#### 3.4.3. Snowboarding 

Observed head injuries among snowboarders ranged from 13% to 50% [78,79], with experienced riders being more represented, possibly due to a higher propensity to take risks [66,68,79], with helmet-wearing snowboarders taking greater risks and thus incurring a higher rate of head injuries than novices [68] or snowboarders that wore no helmets [80]. Not surprisingly, the more complex the attempted activity, the higher the injury rate.

The etiology of injuries on the slopes as well as on level ground sees backwards falls (41%) and forward falls (38%) as the primary cause of head injury caused by loss of control when striking unevenness in the skiing surface [64]. Only three studies detailed the location of the cranial injury, limited to the major bones (frontal, parietal, temporal, occipital, facial) [64,74,75]. Nakaguchi studying head injuries on Japanese ski slopes, found that trauma to the frontal area dominated (53.8%), followed by injuries to the occipital (33.3%) region [75]. On the other hand, Fukuda et al., also studying head injuries on Japanese ski slopes, found that trauma to the occipital area overwhelmingly dominated (62.6%), followed by injuries to the frontal (24.5%) and temporal region (11.7%) [74]. One study, examining the location of the cranial injury *and* the type of fall, found that forward falls impacted the facial and frontal bones, while backward falls impacted the occipital area. Impacts to parietal and temporal bones were negligible [64].

#### 3.4.4. Sledding

Observed head injuries from sledding (tobogganing) ranged from 13% to 58% [62,81], with the incidence decreasing with participant age [82]. The biomechanics of sledding lead to two types of falls; forward falls when the sled strikes an object or an irregularity in the track, and sideways–backwards falls when the rider loses control. Both frontal and occipital trauma can be surmised.

#### 3.4.5. Curling

Injuries incurred while curling, an Olympic winter sport originating in Scotland where a 20 kg stone is slid on an iced surface towards a target (akin to lawn bowls), are predominantly limited to musculoskeletal injury. A single study noted that head injuries which made up 31.7% of all injuries, were all caused by falls. The exact location of the impact points is not discussed apart from singling out the face (6.3%) [83].

### 3.5. Water Sports

This section will look at board surfing, wind and kitesurfing, as well as white-water kayaking and rafting, while sailing will be excluded [84].

#### 3.5.1. Surfing

The epidemiology of head injuries sustained while stand-up surfing is primarily due to the surfer losing control, being thrown off the board, with the board subsequently striking the surfer’s head. A smaller number of head injuries is caused by the head striking the seafloor or reef, impacting the water or hitting the board of a fellow surfer [85,86]. Unlike in other sports, the kinetics of surfing accidents are complex as the body movement is continually modulated by the wave action, with the board acting as an independent variable. It can be posited that the impact sites of trauma are randomly distributed across the body [87]. The board’s movement is somewhat constrained by the leg rope (used to prevent the board from floating away), resulting in increased impact in the facial area (see below). The immersion of the body in the water adds to the dimension of drowning in the case of severe concussion or cranial fractures with associated loss of consciousness. 

A study of surfing-related head trauma requiring hospital admission in Cornwall found that all reported cranial fractures involved the facial bones and the teeth [88]. This was echoed by studies in Australia [89,90,91], Norway [86], and the United States [92,93,94,95], which also found a very high incidence of facial fractures and lacerations. Dimnick et al. argued that the etiology of this injury is greatly determined by the use of leg ropes which tend to be too short and thus constrain the movement of the board in the water [89]. In addition, there is a single individual cases study that documents an impact in the upper parietal [96].

#### 3.5.2. Wind and Kitesurfing

Windsurfing involves the participant standing on a longboard propelled and steered by a board-mounted sail. Kitesurfing, conducted predominantly on snow or water, involves the participant standing on snow or wakeboard while being propelled by a hand-controlled kite or mini sail. Depending on wind strength, wind and kite surfers can attain high speeds. Loss of control leads to surfers falling off the board, primarily falling backwards in the case of windsurfing and sideways in the case of kitesurfing. In addition, contact can occur with the equipment, such as the board (in both sports) or with the mast and boom of the windboard, leading to cranial fractures. Individual cases studies have documented fractures of the frontal sinus [97] and generic facial fractures [98].

#### 3.5.3. White-Water Kayaking and Rafting

Compared to shoulder injuries, head injuries are not common among white-water kayaking and rafting [99] but occur when capsized kayakers or rafters strike the rocks. Facial trauma seems prevalent in the few studies that allow us to separate between cranial and facial injuries [100,101,102,103,104].

### 3.6. Animal-Based Sports

#### Equestrian

The literature review on injury patterns and head injuries sustained in the equestrian sector focused on recreational riders. It specifically excluded papers addressing injuries during professional horse racing events but included studies discussing injuries incurred during agricultural work and rodeos.

Generally, the introduction of helmets has been effective [105] and has seen the incidence of head injury decline from 66% of all accidents [106] to below 20% [107]. The rate of head injuries is not surprising given the standard riding position and the reality that most falling riders are projected head forward and downward [108]. Head injuries decrease with the rider’s age, which may be related to experience [109,110]. This finding is supported by the observation that recreational riders had a higher likelihood of head injury than professional equestrians [111].

Only one equestrian-related study has value for the research question posited in this paper: a French modelling study using multiple rider postures and horse movements found three main impact areas (frontal, parietooccipital, and temporospatial) with the greatest force exerted on the frontal area [112]. 

## 4. Discussion

Setting aside equipment failure and external factors, such as traffic accidents or collisions with fellow participants in an activity, the vast majority of injuries are associated with loss of control and collisions with objects such as trees or boulders. Of all accidents, falls are most common and tend to cause the majority of head injuries. Table 1 provides a synopsis of the documented or inferred directions of falls and how these manifest themselves in broad injury loci on the skull and face.

Given the momentum of most activities, as well as the biomechanical functioning of the equipment in relation to the human body, some general observations can be advanced. Loss of control during mountain biking, for example, tends to result in a forward motion that differentially impacts the face and the frontal bone [38]. Similarly, given the standard riding position, the body’s inertia projects the rider head forward and downward when a horse shies [108]. Loss of control during skateboarding causes the skateboard to slip under the rider’s feet, resulting in a backward motion that differentially impacts the occipital area [18]. This is also the case in snowboarding, with the added dimension that the forward motion can be interrupted by irregularities on the snow surface, resulting in a forward direction of the falls. 

However, some sports, such as climbing, hang-gliding, paragliding, and BASE jumping, exhibit more diffuse etiologies of injuries (Table 1), primarily due to variables other than pure biomechanics and momentum. For example, being hit or struck by a falling object, such as a rock or ice, is the leading cause of head trauma during climbing and mountaineering [19,25,26].

### Protective Potential of Turbans

At present, there are no studies that examine the relative differential protection provided by helmets. As none of the clinical observational studies consulted discusses the exact loci of the trauma, they are unsuited to assess the specific efficacy of helmets or the efficacy of potential helmet alternatives such as turbans. Some studies, however, allow for a general assessment relating to impacts in turbaned and unprotected areas of the cranium (Table 2). All such available studies relate to snow sports injuries, namely downhill skiing and snowboarding. As with most standard helmets, the turban provides no protection for the face, so facio-maxillary injuries can be ignored unless full-face helmets become legally mandated. Injuries affecting areas not protected by turbans range from 3.8% to 13.1% (median 7.7%). Even though a turban leaves 38% of the cranial surface area unprotected, that area appears to be significantly underrepresented as an impact zone.

While there are modelling studies that consider the protective capability of helmets on head trauma, for example, from ballistic impact [113] or blunt impact while cycling [114], only one modelling study has been located that relates to impacts in sports. A French study with 1920 simulations using multiple rider postures as well as horse movements found three main impact areas (frontal, parieto-occipital, and temporospatial) with the greatest force exerted on the frontal area [112]. The angle of the falls and the points of impact are such that the turbaned area coincides with the impact areas, thereby buffering falls.

The question arises, of course, to what extent a turban buffers an impact. Given the overall paucity of data, the attenuation effects of different turbans cannot be considered at this stage of research. The gold standard of a randomized, prospective, controlled trial cannot be performed due to the ethical issues involved [115,116]. In the late 1980s, Sood, examining over 300 cases of head trauma caused by motorcycle accidents in New Delhi, noted that “[t]heir head injury incidence and severity was midway between that of drivers with helmets and without, suggesting that the turban offers some degree of protection” [3], especially in minor accidents. This buffering capacity of a turban depends on the nature of the turban style [5], the thickness of the turban fabric, and the number of windings applied. A Sikh recreational user could, for example, enhance his protection by a longer and thicker turban cloth with additional winding layers. While this would increase buffering, the extra layers would not significantly add to the overall mass of the turban and thus would be extremely unlikely to affect the fall dynamics (e.g., stresses on cervical vertebrae). 

Turbans will have a buffering effect on blunt impact in the frontal, occipital, and temporal or lower parietal regions but will have no protective effect on the sharp impact of the upper part of the cranium, for example, caused by falling stones or ice during climbing.

## 5. Future Research

Given that randomized, prospective, controlled trials are not ethically tenable, future research will rely on observational studies. As was noted in reference to a prior analysis assessing the current discussion on cranial trauma in two-wheeler accidents on the Indian subcontinent [2], formal studies do not specify the exact locus of the cranial trauma, thereby making it impossible to extract relevant data that might constructively indicate whether areas unprotected by turbans are disproportionately affected. Future clinical and forensic work should record injury-related information in more detail, not only regarding the exact location and nature of the impact (blunt, focused, sharp) but also with respect to the associated data on the nature and design of the helmet or skull cap worn. Ideally, this information would also include a record of any damage to the protective headwear [117]. Ancillary data, such as speed of activity, and the resulting impact velocities, should also be recorded where possible. Developing such a comprehensive data set would require sustained coordinated data collection by first responders and clinicians, which may prove too challenging unless conducted under controlled settings such as ski resorts or surf beaches. A detailed recording of the location and nature of the impact, however, is well within the remit of clinicians and forensic practitioners.

## Figures and Tables

**Table 1:** The main direction of fall and broad loci of head injuries according to the available literature.

	Fall Direction			Differentially Preferred Broad Locus of Head Injury					
Sport	For-Ward	Side-Ways	Back-Ward	Down-Ward	Facial	Frontal	Parietal	Temporal	Occipital
Climbing			✓	✓			X *		
**Wheeled sports**									
Mountain/Trail Biking	✓				dom	dom	occ	occ	
Scooters	✓	✓			dom	dom	occ	occ	occ
Skateboarding	✓		✓		dom	dom		occ	dom
Skating	✓		✓		freq	freq			freq
**Aerial sports**									
Hang-Gliding & Paragliding	✓	✓	✓	✓					
Skydiving			✓						dom
BASE jumping	✓	✓	✓	✓					
**Winter sports**									
Downhill skiing	✓	✓		✓	freq	dom			dom
Cross-country skiing	✓				freq	freq			
Snowboarding	✓		✓		freq	freq			freq
Sledding	✓		✓		freq	freq			freq
Curling					freq	freq			
**Water sports**									
Surfing	✓				dom	dom			
Wind and Kitesurfing	✓				dom	dom			
White-water kayaking and rafting	✓				dom	freq			
**Animal-based sports**									
Equestrian	✓			✓	dom	dom	freq	occ	

* includes being struck by falling rocks or ice.—Reported frequency: pre-dominant (dom); frequent (freq); occasional (occ).

**Table 2:** Loci of head injuries.

	Studies Excluding Facial		Studies in Including Facial			
Activity	Turbaned	Unprotected	Turbaned	Unprotected	Facial	Reference
Skateboarding	92.2%	7.8%				[18]
Skiing	91.7%	8.3%				[73]
Skiing	96.2%	3.8%				[74]
Skiing	95.6%	4.8%				[75]
Skiing	(86.9%)	(13.1%)	63.7%	9.6%	26.7%	[64]
Snowboarding	92.5%	7.5%				[75]
Snowboarding	98.8%	1.2%				[74]
Snowboarding	(89.3%)	(10.7%)	63.1%	7.6%	29.1%	[64]

## Data Availability

Not applicable.
